# A Rare Case of Endometrial Adenosarcoma and Its Management: A Case Report and Literature Review

**DOI:** 10.7759/cureus.79970

**Published:** 2025-03-03

**Authors:** Shereen Rafiq, Sofia Ali, Hend Makky Abouzied, Yusra Qamar

**Affiliations:** 1 Radiology, Faisalabad Medical University, Faisalabad, PAK; 2 Medicine, Peninsula Medical School, Plymouth, GBR; 3 Medicine, Thumbay University Hospital, Ajman, ARE; 4 Obstetrics and Gynecology, King George's Medical University, Lucknow, IND

**Keywords:** chemotherapy, diagnosis, endometrial adenosarcoma, hysterectomy, management, uterine malignancy

## Abstract

Endometrial adenosarcoma is a rare and aggressive uterine malignancy composed of both epithelial and stromal components, most commonly affecting postmenopausal women. This case report describes the diagnosis of endometrial adenosarcoma in a 52-year-old woman who presented with abnormal vaginal bleeding. A hysterectomy was performed, and a histopathological examination confirmed the diagnosis. Due to the high risk of recurrence, the patient underwent surgical resection followed by adjuvant chemotherapy. Given the rarity of this tumor, its management presents a significant clinical challenge. This case underscores the diagnostic approach, therapeutic strategies, and the critical role of close follow-up in managing endometrial adenosarcoma.

## Introduction

Adenosarcomas are rare malignancies of the female genital tract, accounting for approximately 5% of uterine sarcomas [1]. In some cases, they may also arise in the ovaries or extrauterine tissue, potentially associated with endometriosis [2]. As a subtype of malignant mixed Müllerian tumors, adenosarcomas consist of malignant epithelial and benign stromal components [3]. Risk factors include prior pelvic radiation, prolonged estrogen exposure, and tamoxifen use, all of which may contribute to their development.

Adenosarcomas exhibit genetic heterogeneity, with 26-28% of cases showing MDM2/CDK4 amplifications. Additional genetic alterations include mutations in the PI3K/AKT/PTEN and Wnt/β-catenin pathways, as well as ATRX, FGFR2, KMT2C, and TP53 mutations, along with TERT and MYLB1 amplifications [4]. While adenosarcomas most commonly affect postmenopausal women, they can also occur in premenopausal individuals.

Although typically indolent, these tumors have a high potential for recurrence and metastasis, particularly to the lungs and lymph nodes. Prognosis depends on factors such as tumor stage, myometrial invasion, and the presence of sarcomatous overgrowth, which is associated with an increased risk of recurrence and metastasis [5]. Given their rarity and diverse histologic features, diagnosis can be challenging and often requires a combination of clinical, radiological, and pathological assessments.

Here, we present a rare case of endometrial adenosarcoma in a 52-year-old woman, highlighting its clinical presentation, diagnostic approach, management, and outcome.

## Case presentation

A 52-year-old woman, G2P2, presented to the Government College University Faisalabad Healthcare Centre with a four-month history of abnormal vaginal bleeding and lower abdominal discomfort. Her medical history included hypertension, but there was no significant history of gynecological malignancy. She had entered menopause two years prior and had no family history of uterine cancer. On physical examination, her abdomen was soft with mild tenderness in the lower pelvic region, but no palpable masses were detected. A pelvic ultrasound, performed via a transvaginal approach, revealed a thickened endometrial stripe and a small submucosal fibroid.

**Figure 1:**
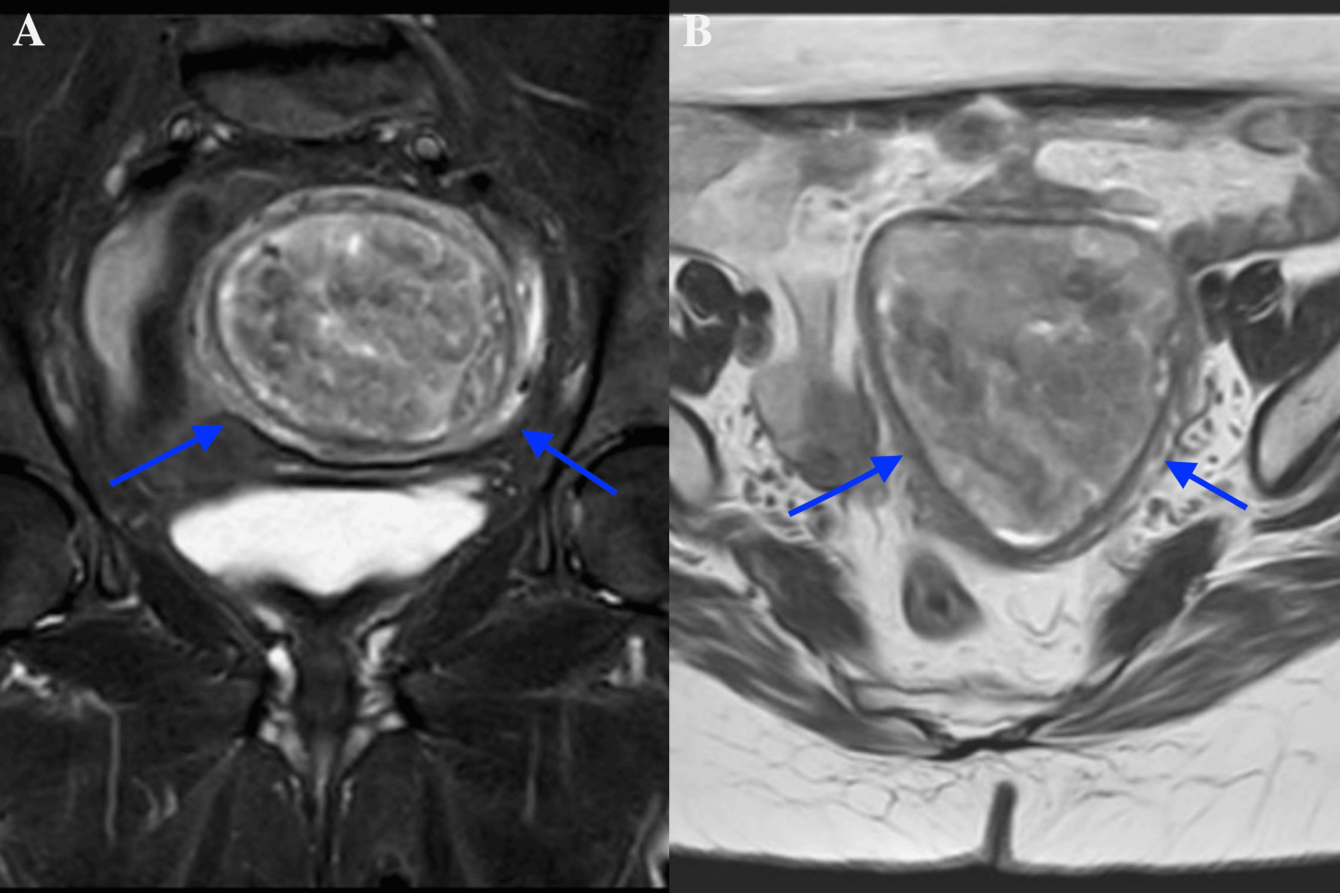
Pelvic MRI showing (A) a coronal view of a large, well-defined endometrial mass with irregular borders and (B) an axial view emphasizing its infiltrative nature, indicative of a malignant process

Initially, the patient received symptomatic treatment, including analgesia for abdominal discomfort and tranexamic acid for abnormal vaginal bleeding. However, due to persistent symptoms despite conservative management, further imaging and biopsy were performed to determine the underlying pathology. An endometrial biopsy revealed a benign endometrial polyp. Subsequently, pelvic MRI demonstrated a well-defined, large mass within the endometrium with irregular borders, suggesting a malignant process (Figure 1a, 1b). Differential diagnoses included endometrial carcinoma, atypical hyperplasia, uterine sarcoma, and, less commonly, a rapidly growing fibroid with degeneration. Given the complexity of the presentation, a hysteroscopy was performed, revealing an irregular, infiltrative mass within the uterine cavity.

**Figure 2:**
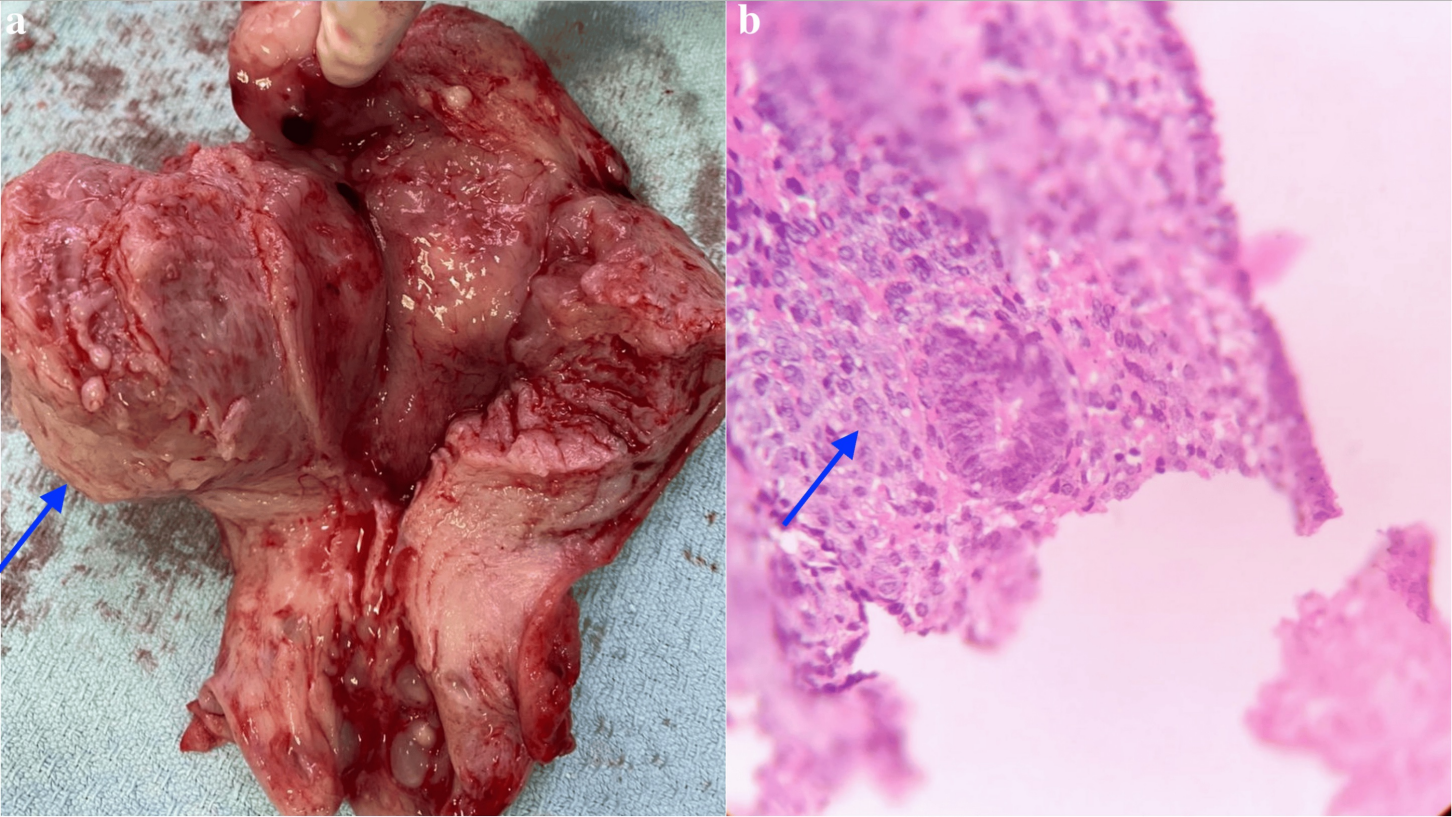
Gross examination showing (A) a mass with hemorrhage and necrosis and (B) histopathology confirming endometrial adenosarcoma with biphasic malignant glandular and benign stromal components

The patient underwent a total abdominal hysterectomy with bilateral salpingo-oophorectomy (BSO). Gross examination of the uterus revealed a 6 cm irregular mass with areas of hemorrhage and necrosis within the endometrial cavity (Figure 2a). The histopathological analysis identified a biphasic tumor composed of benign glandular epithelial and malignant stromal components, confirming the diagnosis of endometrial adenosarcoma (Figure 2b). Immunohistochemical staining demonstrated positive expression of estrogen and progesterone receptors in the stromal component, further supporting the diagnosis.

Given the diagnosis of endometrial adenosarcoma and its high risk of recurrence due to its high-grade nature, the patient was initiated on adjuvant chemotherapy with paclitaxel and carboplatin. Regular follow-up, including pelvic examinations, imaging, and surveillance biopsies, was recommended to monitor for recurrence. The patient tolerated both surgery and chemotherapy well. Routine CT scans and pelvic ultrasounds showed no evidence of metastatic disease at the six-month follow-up. At her most recent evaluation, 18 months post-surgery, there were no signs of recurrence, and she remains under observation with plans for continued surveillance every six months.

## Discussion

We report a rare case of endometrial adenosarcoma, a malignancy composed of benign glandular and low-grade malignant stromal components. Uterine sarcomas account for only 3% of all uterine malignancies, with uterine adenosarcomas comprising 5-10% of cases within this category [6]. Endometrial adenosarcoma, a subtype confined to the endometrial lining, can occasionally invade the myometrium or Müllerian duct remnants. It may also affect other gynecologic sites, including the cervix, ovary, fallopian tube, and vagina [6]. While uterine adenosarcoma has a lower malignant potential compared to carcinosarcoma, its rarity limits the available literature on management and outcomes [7].

A retrospective analysis found that most patients with endometrial adenosarcoma are postmenopausal Caucasian women, with median ages of 54, 56, and 58, as seen in this case [7-9]. Abnormal uterine bleeding is the most common presenting symptom, reported in 65% of cases in a study by Carroll et al. [7]. Less frequently, 10% of patients present with a pelvic mass, which in this case was not palpable but was visible on MRI [9]. Rare presentations include an enlarged uterus, protruding tissue from the external cervical os on speculum examination, and recurrent polyps on pelvic ultrasound [9]. Other symptoms include uterine enlargement, prolapse, vaginal discharge, dysmenorrhea, infertility, and abdominal distension due to ascites [10]. Tamoxifen use has been weakly associated with adenosarcomas of the reproductive tract, while extrauterine cases have been linked to endometriosis [6].

MRI is the preferred imaging modality for endometrial adenosarcoma due to its superior soft tissue resolution compared to ultrasound or CT. It typically reveals a well-defined polypoid mass arising from the endometrium and extending into the internal os, often accompanied by necrosis, hemorrhage, and small cystic areas [11]. MRI effectively evaluates myometrial invasion, while diffusion-weighted imaging (DWI) signals vary depending on the proportion of benign epithelium versus malignant mesenchymal components [10]. Common findings also include uterine enlargement and myometrial thinning [11]. PET and CT scans assist in detecting metastases and recurrence [10]. The correlation between MRI and histopathology enhances diagnostic accuracy, with imaging features such as polypoid morphology, cystic changes, periglandular stromal cuffing, and mesenchymal proliferation aligning with microscopic findings. Variability in DWI signal intensity reflects tumor heterogeneity, where benign epithelial areas appear hypointense and malignant stromal proliferation presents as hyperintense, aiding in differentiation from other uterine neoplasms [10].

Given the nonspecific presentation and imaging characteristics of this malignancy, histopathological biopsy is essential for diagnosis. Similar to phyllodes tumors of the breast, endometrial adenosarcomas exhibit a leaf-like architecture on low-power microscopy due to malignant stromal compression of the benign epithelium [6]. The malignant stroma lines large, dilated cystic structures, displaying characteristic periglandular cuffing of stromal cells. The epithelial component includes endometrioid cells with squamous and mucinous metaplasia, while the stromal component commonly consists of low-grade spindle cell sarcoma with myxoid foci and mitotic activity, particularly in periglandular cuffing regions [6]. Diagnosis requires identifying mitotic figures (MFs) in malignant stromal components, with a mean of 9 MF/10 HPF typically observed, although only 2 MF/10 HPF are required for diagnosis [12]. Sarcomatous overgrowth, a rare but aggressive feature, is defined as pure malignant stromal proliferation comprising at least 25% of the tumor [12].

Distinguishing endometrial adenosarcoma from adenofibroma presents another diagnostic challenge due to overlapping histological features. Zaloudek and Norris identified key differentiating factors, including the number of MFs, degree of mesenchymal atypia, and presence of malignant heterologous elements [13]. The International Federation of Gynecology and Obstetrics (FIGO) and the American Joint Committee on Cancer have established the FIGO Surgical Staging system for uterine adenosarcoma, categorizing stages from I to IVB.

Immunohistochemistry supports diagnosis by distinguishing glandular and stromal components. Typically, both components express estrogen and progesterone receptors; however, in this case, only the malignant stromal component showed immunoreactivity [6]. Ki-67 expression differs across adenosarcoma subtypes, including those with sarcomatous overgrowth and carcinomas, but overlaps with endometrial polyps, endometriosis, and adenofibroma [14]. Endometrial adenosarcoma generally exhibits weak p53 expression and strong CD10 reactivity, whereas adenosarcomas with sarcomatous overgrowth and carcinomas show reduced CD10 staining [12]. Epidermal growth factor receptor negativity further differentiates adenosarcoma from sarcomatous overgrowth and carcinomas, aligning it more closely with endometrial polyps and endometriosis [12]. Mesenchymal markers such as CD34 and desmin smooth muscle actin (SMA) show variable expression in the stromal component, with SMA and desmin being more prevalent [6].

The primary treatment for endometrial adenosarcoma is complete surgical removal, often followed by adjuvant chemotherapy due to the risk of carcinosarcoma transformation. Hysterectomy is the standard surgical approach, although fertility-preserving local excision may be considered in select reproductive-age patients [14]. BSO is recommended for cases with myometrial invasion or sarcomatous overgrowth due to the high incidence of adnexal involvement and hormone receptor expression [14]. Standard chemotherapeutic regimens include doxorubicin, cyclophosphamide, dacarbazine, and vincristine to prevent metastasis [15]. The role of lymphadenectomy remains controversial, as only 2.9% of endometrial adenosarcomas exhibit lymph node involvement [14]. While some studies suggest minimal survival benefit, a retrospective analysis of 994 cases and a systematic review of 230 cases found lymph node involvement to be the strongest predictor of progression-free survival, surpassing myometrial invasion and sarcomatous overgrowth [10]. If lymph nodes are enlarged, excision and biopsy are recommended.

The overall survival rate for endometrial adenosarcoma ranges from 60% to 87%, with prognosis influenced by staging, myometrial invasion, sarcomatous overgrowth, and lymph node involvement [10]. However, due to the low prevalence of adenosarcoma, standardized treatment options for metastatic or recurrent cases remain limited. Current approaches include chemotherapy, palliative radiation, surgery, and hormonal therapy, with significant variation in medication regimens [16].

Recent advancements in endometrial adenosarcoma treatment focus on targeted and immunotherapeutic strategies. Hormonal therapies, such as aromatase inhibitors and selective estrogen receptor modulators, are being explored due to tumor hormone receptor expression [17]. Ongoing research and clinical trials aim to refine chemotherapy regimens and improve outcomes in metastatic and recurrent cases.

## Conclusions

Endometrial adenosarcoma is a rare and potentially aggressive uterine malignancy, often presenting a diagnostic challenge due to its mixed histological features. This case highlights the critical role of MRI and hysteroscopy in early detection, with complete surgical resection and adjuvant chemotherapy as the primary treatment strategies. In advanced or recurrent cases, systemic therapies, including chemotherapy or hormonal treatment, may be necessary. Given the risks of recurrence, metastasis, and treatment-related complications, long-term surveillance is essential. A multidisciplinary approach remains crucial for optimizing patient outcomes and improving prognosis.
